# Different photosynthetic inorganic carbon utilization strategies in the heteroblastic leaves of an aquatic plant *Ottelia ovalifolia*

**DOI:** 10.3389/fpls.2023.1142848

**Published:** 2023-03-24

**Authors:** Zuying Liao, Pengpeng Li, Jingzhe Zhou, Wei Li, Hong Sheng Jiang

**Affiliations:** ^1^ Aquatic Plant Research Center, Wuhan Botanical Garden, Chinese Academy of Sciences, Wuhan, China; ^2^ University of Chinese Academy of Sciences, Beijing, China; ^3^ Hainan Key Laboratory for Sustainable Utilization of Tropical Bioresources, School of Life Sciences, Hainan University, Haikou, China; ^4^ Research Center for Ecology, College of Science, Tibet University, Lhasa, China

**Keywords:** *Ottelia ovalifolia*, heteroblastic plant, bicarbonate use, C4, carbon isotope ratio

## Abstract

The leaves of the heteroblastic aquatic plant *Ottelia ovalifolia* faces submerged and aerial environments during its life history. However, the acclimation of the submerged leaves and floating leaves to these two environments in morphology, physiology, and biochemistry remain unclear. In the present study, we investigated the acclimation of the CO_2_-concentrating mechanisms in these two types of leaves. We found that the submerged leaves were longer, narrower, and thinner than the floating leaves, which increased the specific surface area of the leaves and lead to better absorption of the inorganic carbon underwater. Meanwhile, the floating leaves absorbed atmospheric CO_2_ directly through the stomata to acclimate to the aerial environment. Both the leaf types had the ability to use ${\text{HCO}}_{3}^{-}$ , but the capacity in submerged leaves was stronger than that in floating leaves. The extracellular carbonic anhydrase and anion exchanger were responsible for the ${\text{HCO}}_{3}^{-}$ use in both types of leaves. The higher ratio of chlorophyll a/b and content of anthocyanin in floating leaves than that in submerged leaves indicated that the acclimation of aerial and submerged photosynthesis depended on changes in the photosynthetic pigments. Based on the stable carbon isotope ratio, key enzyme activities of the C4 pathway indicated that submerged leaves might have the ability to perform C4 metabolism while floating leaves only performed C3 metabolism. In summary, *O. ovalifolia* acclimates to submerged and aerial environments through changes in morphology, physiology, and biochemistry during different growth stages.

## Introduction

The distribution of plants is driven by the ability of plants to disperse, establish, and maintain in their specific environments (Cavalli et al., 2012). Species that acclimate well to different environments are better able to take up positions in habitats and may displace other species or even alter the habitats (Cavalli et al., 2012). Due to the high transport resistance, high water pH and the external boundary layer (Raven, 1970; Maberly and Madsen, 1998), CO_2_ shortage is considered to be one of the limiting factors for photosynthesis of submerged plants (Maberly and Madsen, 1998) that play an important role in aquatic ecosystem, as they provide food, habitat and shelter for invertebrates and fish (Chambers et al., 2008). Although CO_2_ is the preferred carbon source for submerged photosynthesis, it only contributes to a very small proportion of dissolved inorganic carbon (DIC) and ${\text{HCO}}_{3}^{-}$ is the main form of DIC in most natural water body. It is generally believed that the acquisition and utilization of limited resources by plants is highly competitive, which have an important effect on plants distribution (James et al., 1999). Adapting to the shortage of CO_2_ under water, submerged plants develop several CO_2_ concentrating mechanisms (CCMs) that enrich the available concentration of CO_2_ at the active site of ribulose 1,5-bisphosphate carboxylase/oxygenase (Rubisco) in physiological and biochemical response including ${\text{HCO}}_{3}^{-}$ use, crassulacean acid metabolism (CAM)-like and C4 (or C4-like) metabolism (Klavsen et al., 2011). Abundant ${\text{HCO}}_{3}^{-}$ in field freshwater can serve as an additional carbon source for photosynthesis in about 44% of tested aquatic plants and reduce photorespiration under low CO_2_ availability (Maberly and Gontero, 2017; Yin et al., 2017; Iversen et al., 2019; Jiang et al., 2021; Li et al., 2021). Two mechanisms of ${\text{HCO}}_{3}^{-}$ use have been studied in detail in microalgae, macroalgae, seagrasses and a small range of freshwater aquatic plants (Giordano et al., 2005; Axelsson et al., 1995; Huang et al., 2020a; Jiang et al., 2021): I) Extracellular carbonic anhydrase (CAext) catalyzes the conversion of ${\text{HCO}}_{3}^{-}$ into CO_2_ that enters the cell and participates in photosynthesis. This mechanism of ${\text{HCO}}_{3}^{-}$ use was widespread in aquatic plants (Huang et al., 2020a); II) ${\text{HCO}}_{3}^{-}$ is directly absorbed *via* an anion exchanger (AE) protein located at the plasmalemma. These two mechanisms are not mutually exclusive but complementary in some species, and they will be responsible for the ${\text{HCO}}_{3}^{-}$ uptake under different DIC environments that could cope with the variation of DIC in the natural waterbody (Axelsson et al., 1995; Huang et al., 2020a; Jiang et al., 2021). Both CAM and C4 perform the first step of carbon fixation with phosphoenolpyruvate carboxylase (PEPC) whose active is temporal separation for CAM plants or spatial separation for C4 plants (Maberly and Gontero, 2017). CAM is a malleable metabolism in aquatic plants compared to land plants (Bowes and Salvucci, 1989) and 9% of aquatic plant perform CAM (Maberly and Gontero, 2018). For instance, CAM could be induced under low CO_2_ conditions, but not under high CO_2_ conditions in the submerged macrophyte *Ottelia alismoides* (Shao et al., 2017). On the other hand, C4 metabolism was found in about 4% of the tested aquatic plants (Maberly and Gontero, 2018) and it typically involves a PEPC catalyzing phosphoenolpyruvate (PEP) and ${\text{HCO}}_{3}^{-}$ to form a 4-carbon acid, which is then cleft by a decarboxylase to produce CO_2_ in the vicinity of Rubisco (Clement et al., 2016). C4 metabolism in terrestrial plants is often associated with Kranz-type anatomy, but some submerged plants lacking Kranz-type structures also possess C4 metabolism, such as *Hydrilla verticillata*, *Egeria densa*, and *O. alismoides* (Bowes et al., 2002; Lara et al., 2002; Zhang et al., 2014). Compared to CCMs in terrestrial plants that are adapted to high temperature and drought, aquatic plants develop CCMs most widely in low CO_2_, high ${\text{HCO}}_{3}^{-}$ , high pH, and high light environments (Maberly and Gontero, 2017) and perform changes in morphology and physiological function.

The leaf is the main photosynthetic organ in higher plants and its morphology and anatomy could also reflect the influences of their growth environments (Vogelmann and Gorton, 2014). For instance, the heteroblastic species, *O. cordata*, grew submerged leaves at the juvenile stage and floating or aerial leaves at the adult stage (Wang et al., 2022). Floating and aerial leaves are directly exposed to the air, and they have similar anatomical structures to terrestrial plants, with palisade tissue and spongy tissues differentiated, and stomata in the upper and/or lower epidermis (Han et al., 2021; Horiguchi et al., 2021; Wang et al., 2022). The morphology and anatomical structure of submerged leaves indicate that they are adapted to water habitat: submerged leaves are thin, with normally two to three layers of cells and their morphologies are whorled, dissected whorled, dissected, linear, linear strap-like and filiform, which could increase the specific surface area and better exploit DIC in the water (Shao et al., 2017; Maberly and Gontero, 2018; Horiguchi et al., 2021; Wang et al., 2022).

*Ottelia ovalifolia* (R. Br.) Rich, a species from the Hydrocharitaceae and native to Australia (Paice et al., 2017a), is a heteroblastic plant with submerged leaves at the juvenile stage and floating leaves at the adult stage (Arber, 1919; Paice et al., 2017b). This species was the first invasive aquatic plant recorded in New Zealand and had a large biomass in slow water, affecting the drinking water of livestock (McCullough, 1997). Previous study also found that the aquatic plant *O. alismoides* from the same genus has invaded Europe (Hussner, 2012). In addition, the aquatic plants in the Hydrocharitaceae, the same family of *O. ovalifolia*, such as *E. densa* invaded East Asia and Europe (Hussner, 2012; Wang et al., 2016), and *H. verticillata* invaded America (Sousa, 2011), they all showed highly invasive capacity in East Asia, Europe, North America and New Zealand (McCullough, 1997). It is interesting to find that these aquatic plants were reported to possess at least one of the CCMs, and previous studies showed that the ability of CCMs has an impact on aquatic plant distribution and species composition in the CO_2_ shortage natural water (Maberly and Gontero, 2018; Iversen et al., 2019; Jiang et al., 2021). Current studies on *O. ovalifolia* mainly focus on the factors influencing plant distribution and the potential of submerged plants to support food webs (Paice et al., 2017a; Paice et al., 2017b), but little is known of its CCMs. Therefore, in the present study, we will investigate the CCMs on *O. ovalifolia* and we hypothesized that both submerged and floating leaves of *O. ovalifolia* had CCMs: ${\text{HCO}}_{3}^{-}$ use, C4 and CAM.

## Materials and methods

### Plant materials

*O. ovalifolia* was collected from the resource nursery of Wuhan Botanical Garden, Chinese Academy of Sciences. Three growth stages were found in resource nursery: juvenile growth stage (with only submerged leaves), transition growth stage (with submerged and floating leaves), and adult growth stage (with only floating leaves). In this study, submerged leaves were selected from healthy plants at the juvenile growth stage to avoid the effects from floating leaves and floating leaves were selected from healthy plants at the adult growth stage to exclude the effect from submerged leaves. The experiments were conducted from June to September 2021. The pH of the *in-situ* water was measured by a hand-held pH meter with an accuracy of ± 0.05 (az8886, AZ Instrument, China) at 9:30, and the alkalinity of the water was determined by the Gran titration with 0.1 M HCl (Zhang et al., 2014). The chemical characteristics of inorganic carbon *in-situ* water were shown in **Table 1**.

**Table 1:** Inorganic carbon chemistry in the growth culture in the field.

pH	Alkalinity (mequiv L^−1^)	CO_2_ (mM)	${\text{HCO}}_{3}^{-}$ (mM)
7.59 (7.41–7.7)	1.11 (1.09–1.13)	0.055 (0.041–0.078)	1.102 (1.086–1.121)

### Morphology, anatomy, ultrastructure and photosynthetic pigments

Leaf morphology was described as fresh weight, area, length, width, leaf mass per area (LMA) and aspect ratio. Fresh leaves were photographed with a digital camera (Huawei Mate30pro, China), and the length, width and area of five independent individual leaves were measured using Image J software 1.50 (NIH, USA). The stomatal density of the leaf epidermis of five independent individual leaves was observed under light microscopy (PH100-341L-PL, Purkinje General, China). After cleaning, the leaf fragments (2 cm × 1 cm, length × width) were fixed in 2 mL FAA fixative solution (FAA, 50% ethanol: glacial acetic acid: 40% formaldehyde = 90:5:5, v/v). Subsequently, the paraffin sections were obtained from the fixed slides (Khan et al., 2022) and then the cells length, width and area were measured by Image J software. Fragments of floating and submerged leaves (3 mm × 3 mm, length × width) were fixed overnight in 2.5% glutaraldehyde solution prepared with 0.1M phosphate buffer (pH7.4) at 4 °C and then post-fixed in 1% OsO_4_ solution for 2.5h at 4 °C (Farnese et al., 2017). Ultrathin sections (72 nm) were obtained on a Leica EM UC7 ultramicrotome, and the operation procedure was referred to Han et al. (2020). Chlorophyll content was measured by a spectrophotometer (TU-1810PC, Purkinje General, China) at the absorbance of 470, 665 and 649 nm after 0.1 g fresh weight leaves were extracted in 5 mL 95% ethanol at 4 °C for 24 hours (Shao et al., 2017), each type of leaf had 15 replicates. The anthocyanin content of 0.1 g fresh weight leaves was performed as described by (Solfanelli et al., 2006). Anthocyanin content was expressed by A535/g (FW), each type of leaf had 5 replicates.

### pH-drift experiments

The ability of ${\text{HCO}}_{3}^{-}$ use of floating and submerged leaves was assessed by pH-drift experiments as described in a previous study (Zhang et al., 2014). In addition, previous studies have shown that some *Ottelia* plants are able to utilize ${\text{HCO}}_{3}^{-}$ through CAext and AE (Huang et al., 2020a; Jiang et al., 2021; Wang et al., 2022). The CAext inhibitor acetazolamide (AZA) and AE inhibitor 4,4’-diisothio‐cyanatostilbene-2,2’-disulfonate (DIDS) (Beer et al., 2002) were added to the pH-drift system to verify the ${\text{HCO}}_{3}^{-}$ utilization pathway in *O. ovalifolia*. One treatment without inhibitors was named control, one treatment containing 0.2 mM AZA was named AZA, and the other treatment containing 0.3 mM DIDS was named DIDS. After 24 h illumination determined the endpoint pH in each treatment with eight replicates for the control and five replicates for AZA and four replicates for DIDS treatments.

### Measurement of stable ^13^C isotope

Four submerged leaves and five floating leaves were oven dried and then the stable ^13^C isotope was determined as described in previous studies (Jiang et al., 2021; Wang et al., 2022) by using a Delta Plus Advantage isotope ratio mass spectrometer (Thermo Fisher Scientific, Bremen, Germany). For the δ^13^C DIC analysis of *in-situ* water, the method of Li et al. (2008) was used, with a precision of 0.1 ‰. Isotope data was shown in the conventional delta notion (‰) versus Vienna Pee Dee Belemnite (V-PDB).

### Measurement of photosynthesis enzymes activities

The key enzymes (Rubisco, PEPC and pyruvate phosphate dikinase (PPDK)) activities of carboxylation and decarboxylation in C3 and C4 metabolism of five individuals in submerged and floating leaves were measured by using the method described in Zhang et al. (2014).

### Measurement of acidity

Diurnal variation of titratable acidity was calculated as the difference between the minimum (samples collected at dusk: 18:00) and maximum (samples collected at dawn: 4:30) titratable acidity per unit of fresh mass (Zhang et al., 2014). Each treatment had five replicates.

### Statistical analysis

Data were shown as mean and standard deviation (SD). The statistical analyses were performed in software R version 4.0.5. In the absence of special instructions, we generally used t-test for difference analysis between submerged and floating leaves. Analysis of pH drift experiments without (control) or with inhibitors (AZA, DIDS) were determined by a one-way ANOVA with a Tukey’s HSD test when *p<0.05*.

## Results

### Morphological, anatomic and photosynthetic features

**Figure 1:**
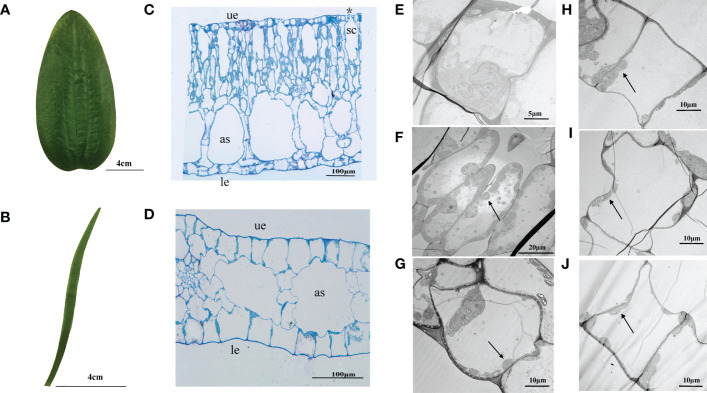
Leaf morphology and anatomical structure in transverse sections of *O. ovalifolia*. **(A)** Morphology of floating leaf; **(B)** Morphology of submerged leaf; **(C)** Transection structure of floating leaf; **(D)** Transection structure of submerged leaf; **(E)** TEM image showing the ultrastructure of upper epidermal cell in floating leaf; **(F)** TEM image showing the ultrastructure of mesophyll cell in floating leaf; **(G)** TEM image showing the ultrastructure of lower epidermal cell in floating leaf; **(H)** TEM image showing the ultrastructure of upper epidermal cell in submerged leaf; **(I)** TEM image showing the ultrastructure of mesophyll cell in submerged leaf; **(J)** TEM image showing the ultrastructure of lower epidermal cell in submerged leaf. as, air space; le, lower epidermis; sc, stomatic chamber; ue, upper epidermis; asterisk indicates stoma; black arrows indicate chloroplasts.

The morphology of *O. ovalifolia* leaves was dramatically different between the floating and submerged leaves. The floating leaves were oval or broadly cordate shape while the submerged leaves were linear (**Figures 1A, B**). Significant differences in leaf length, leaf width, leaf length/width, leaf area and LMA were found between floating and submerged leaves (**Table 2**). The floating leaves were wider, heavier and had a larger area and LMA (**Table 2**). The submerged leaves, however, were longer and had a larger leaf length/width (**Table 2**).

**Table 2:** Morphological, anatomic, photosynthetic and carbon isotope characters in floating and submerged leave of *O. ovalifolia*.

Parameters	Floating leaf	Submerged leaf
Leaf length (cm)	14.9 ± 1.4 b	21.2 ± 3.51 a
Leaf width (cm)	5.86 ± 0.63 a	0.54 ± 0.08 b
Leaf length/width	2.57 ± 0.27 b	39.79 ± 4.84 a
Leaf area (cm^2^)	70.0 ± 13.4 a	10.2 ± 2.3 b
Leaf FW (g)	2.49 ± 0.38 a	0.12 ± 0.03 b
LMA g m^-^²	358.1 ± 19.9 a	111.2 ± 14.2 b
Upper epidermis cell length (μm)	29.6 ± 5.1a	38.0 ± 2.3 a
Upper epidermis cell width (μm)	17.8 ± 4.9 a	12.8 ± 3.7 a
Lower epidermis cell length (μm)	42.45 ± 6.0 a	36.4 ± 7.5 a
Lower epidermis cell width (μm)	23.0 ± 4.9 a	12.0 ± 3.0 a
Upper epidermis cell area (mm^2^)	767 ± 143 a	483 ± 100 a
Lower epidermis cell area (μm^2^)	1156 ± 233 a	434 ± 153 a
Upper mesophyll cell length (μm)	65.1 ± 8.5 a	38.8 ± 11.8 b
Upper mesophyll cell width (μm)	17.2 ± 6.2 a	15.1 ± 0.7 a
Upper mesophyll cell area (μm^2^)	1381 ± 275 a	514 ± 98 a
Lower mesophyll cell length (μm)	62.8 ± 11.1a	38. 8 ± 11.8 b
Lower mesophyll cell width (μm)	27.2 ± 7.1 a	15.1 ± 0.7 b
Lower mesophyll cell area (μm^2^)	2023 ± 9883 a	5143 ± 983 a
Air space area (μm^2^)	14361 ± 38081 a	9850 ± 5559 a
Stomatic chamber area (μm^2^)	13561 ± 4711	
Stomatal density surface (individual mm^2^)	65.4 ± 14.5	
Chl−a (mg g^-1^)	0.60 ± 0.11 a	0.60 ± 0.06 a
Chl−b (mg g^-1^)	0.21 ± 0.04 b	0.25 ± 0.04 a
Chl−a/b	2.80 ± 0.16 a	2.39 ± 0.14 b
Car (mg g^-1^)	0.05 ± 0.02 a	0.05 ± 0.02 a
anthocyanin content (A535 g^-1^)	10.10 ± 1.84 a	1.07 ± 0.29 b
Leaf δ ^13^C (‰)	−30.07 ± 0.54 a	−25.33 ± 0.51 b
DIC δ ^13^C (‰)	−10.92 ± 2.14	−10.92 ± 2.14

Data were expressed as mean ± standard deviation. Different letters indicated significant difference between floating and submerged leaves by t-test (p<0.05).

FW, fresh weigh; LMA, leaf mass per area; Chl-a, b, chlorophyll a and chlorophyll b.

The anatomical structure of floating and submerged leaves was shown in **Figures 1C, D**. The observation of the transverse section showed that the upper and lower epidermal cells of both floating and submerged leaves were composed of single-layered rectangular cells, neatly and closely arranged (**Figures 1C, D**). The mesophyll cells of floating leaves were differentiated into palisade tissue and spongy tissue (**Figure 1C**), while the submerged leaves were not (**Figure 1D**). The length of mesophyll cells and the upper epidermis of floating leaves was significantly larger than that of submerged leaves (**Table 2**). Only the length of upper, the length of lower mesophyll cells and the width of lower mesophyll cells in floating leaves were significantly higher than in submerged leaves (**Table 2**). There were air spaces between the mesophyll cells of floating and submerged leaves, however, no significant difference was found in the air space transection area of floating and submerged leaves (**Table 2**). In the floating leaves, chloroplasts were only found in lower epidermal cells and mesophyll cells, but did not occur in the upper epidermal cells (**Figures 1E–G**). However, chloroplasts were found in both upper and lower epidermal cells, and mesophyll cells of submerged leaves (**Figures 1H–J**). Stomata appeared on the upper surface, and the stomata density was 65 individuals per mm^2^, but there were no stomata on the upper and lower surfaces of the transverse sections of the submerged leaf (**Table 2**; **Figure S1**).

No significant difference in Chl-a content was found between floating and submerged leaves (**Table 2**). However, the Chl-b content in submerged leaves was significantly higher than that in floating leaves (**Table 2**) and the Chl-a/b in floating leaves weas significantly higher than that in submerged leaves (**Table 2**). In contrast to Chl-b, the content of anthocyanin in floating leaves was significantly higher than that in submerged leaves (**Table 2**). The content of anthocyanin in floating leaves was significantly lower than that in submerged leaves (**Table 2**).

### δ^13^C isotope values

The δ^13^C isotope of DIC in water was –10.92 ‰, and the δ^13^C isotope in floating leaves (–30.07‰) was significantly lower than that in submerged leaves (–25.33‰) (**Table 2**). On the basis of the isotope fractionation between ${\text{HCO}}_{3}^{-}$ and CO_2_ (Mook et al., 1974), when the δ^13^C isotope of DIC was –10.92 ‰, the value of δ^13^C for ${\text{HCO}}_{3}^{-}$ calculated to be –10.5‰, while the value of δ^13^C for dissolved free CO_2_ was –18.4‰.

### pH-drift experiments

**Figure 2:**
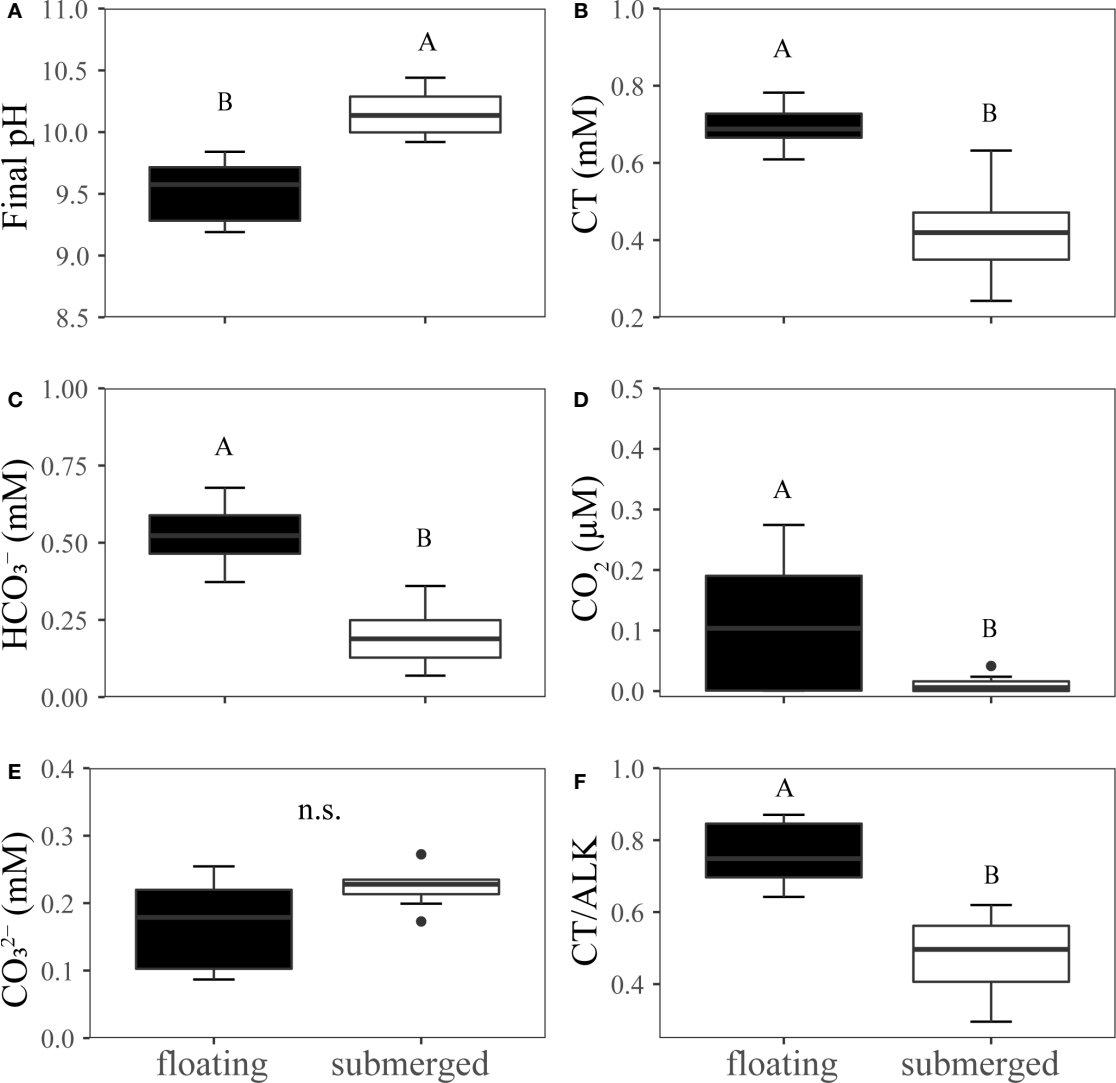
Experimental analysis of pH drift in floating and submerged leaves of *O. ovalifolia*. **(A)** Final pH. **(B)** CT. **(C)** ${\text{HCO}}_{3}^{-}$ . **(D)** CO_2_. **(E)** ${\text{CO}}_{3}^{2-}$ . **(F)** CT/Alk. CT, total inorganic carbon; Alk, alkalinity. n.s., not significant. n=8. The different uppercase letters indicate significant differences (p < 0.05) between floating leaf and submerged leaf.

**Figure 3:**
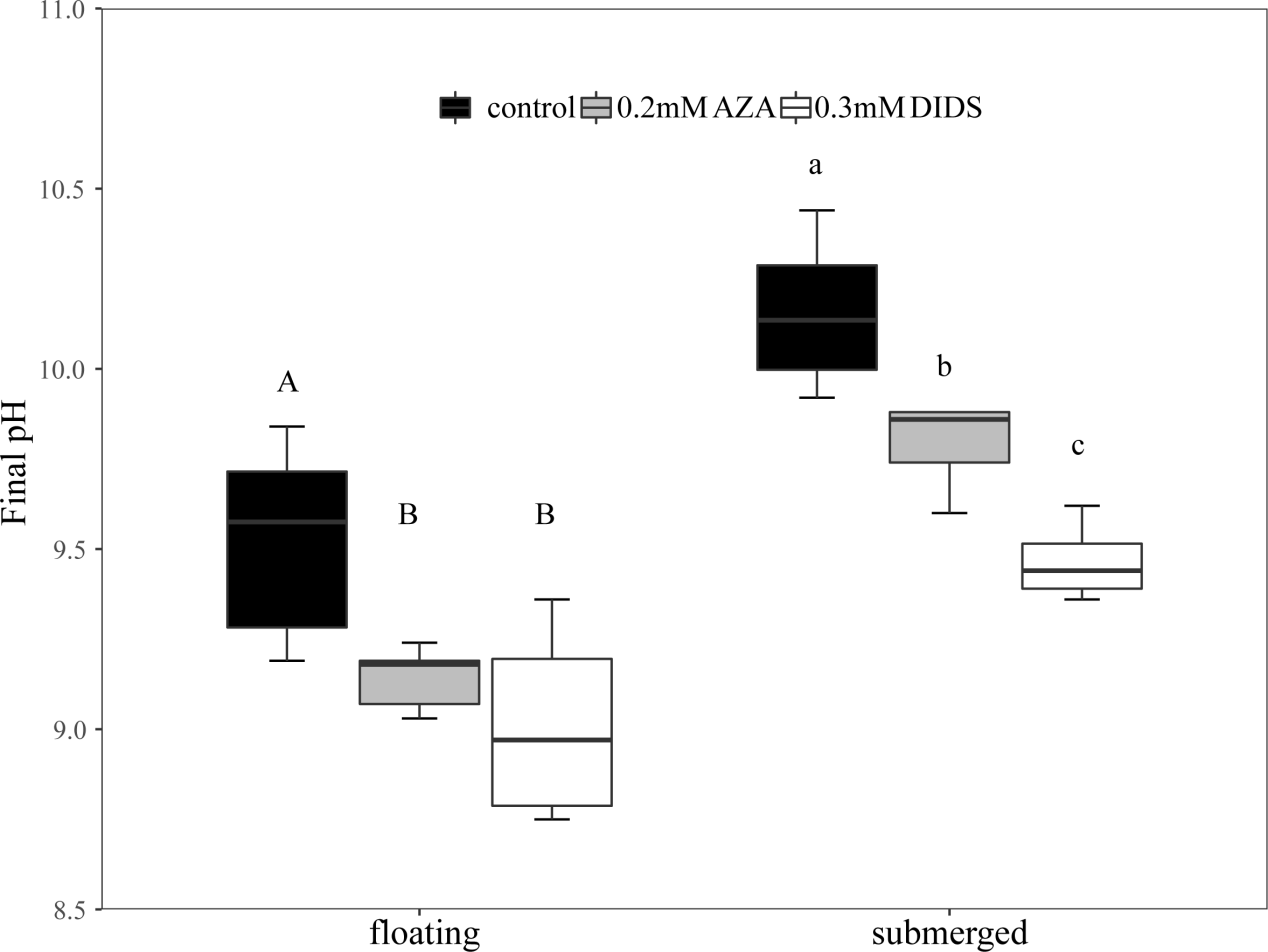
Final pH in the inhibitors adding pH-drift experiments. The different uppercase letters in floating leaves indicate significant differences among control, 0.2 mM AZA and 0.3 mM DIDS. The different lowercase letters in submerged leaves indicate significant differences among control, 0.2mM AZA and 0.3 mM DIDS. n= 4~8.

The average final pH in submerged leaves was 10.2 and was significantly higher than that in floating leaves (9.7) (**Figure 2A**). The remained concentration of CT, ${\text{HCO}}_{3}^{-}$ , CO_2_ and CT/Alk in the pH-drift system of floating leaves was significantly higher than that of submerged leaves (**Figures 2B–D, F**). The concentration of ${\text{CO}}_{3}^{2-}$ was not significantly different between floating and submerged leaves (**Figure 2E**). The inhibitors adding pH-drift experiments showed that 0.2mM AZA and 0.3mM DIDS significantly decreased the final pH both in floating and submerged leaves (**Figure 3**).

### Photosynthesis enzymes activities

**Figure 4:**
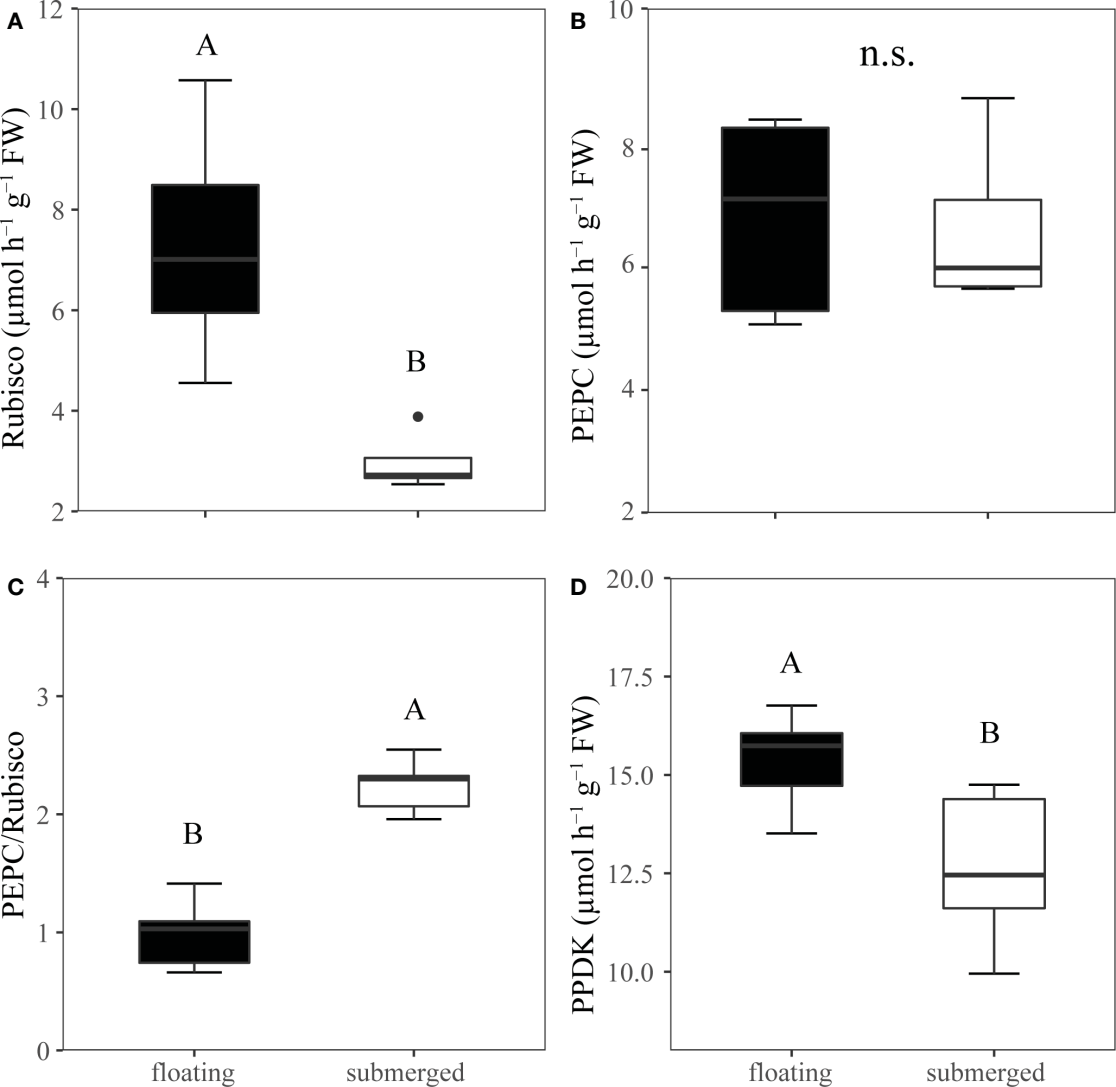
Comparison the crude extracts of C3 and C4 metabolic enzyme activities from *O. ovalifolia* in floating and submerged leaves. **(A)** Rubisco, ribulose 1,5-bisphosphate carboxylase–oxygenase. **(B)** PEPC, PEP carboxylase. **(C)** Ratio of PEPC to Rubisco activity. **(D)** PPDK, pyruvate phosphate dikinase. n=5. The different uppercase letters indicate significant differences (p < 0.05) between floating leaf and submerged leaf. n.s., not significant.

Rubisco activity, a key enzyme of the Calvin cycle, was significantly higher in floating leaves than that in submerged leaves (**Figure 4A**). However, the activity of another carboxylase, PEPC, was not significantly different between floating leaves and submerged leaves (**Figure 4B**). As a consequence, the ratio of PEPC/Rubisco activities in submerged leaves (2.24) was significantly higher than that in floating leaves (0.988) (**Figure 4C**). Similarly, the activity of PPDK performed the same pattern as PEPC based on the fact that the activity of PPDK in floating leaves was significantly higher than that in submerged leaves (**Figure 4D**).

### Titrated acidity

**Figure 5:**
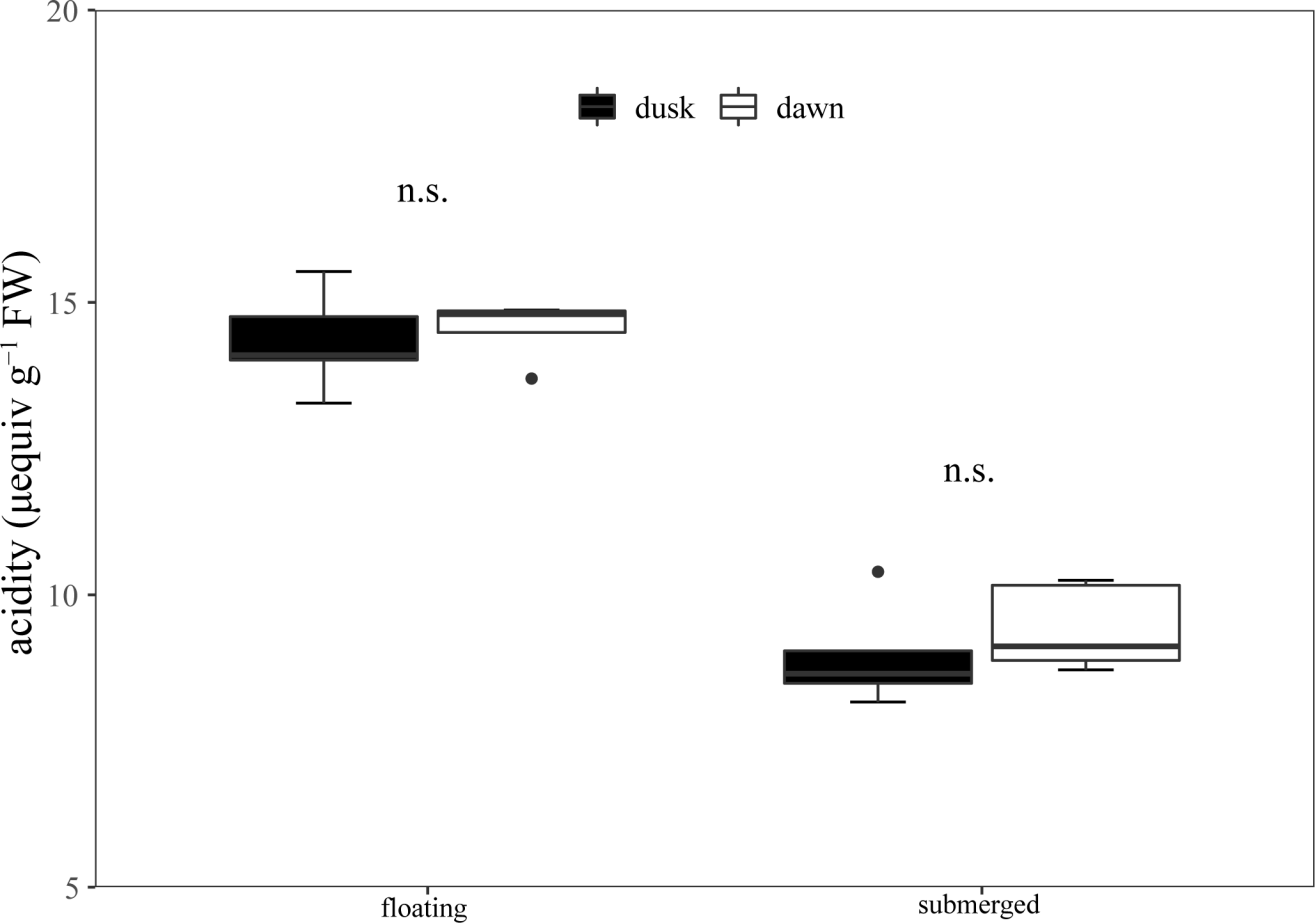
Diel changes of titrated acidity in the floating and submerged leaves of *O. ovalifolia*. n.s.: not significant. n = 5.

The titratable acidity at dusk and dawn was 14.34 and 14.54 μequiv g^−1^ FW in floating leaves, and there was no significant difference between dusk and dawn (**Figure 5**). Additionally, the titratable acidity of submerged leaves at dusk and dawn was 8.95 and 9.42 μequiv g^−1^ FW respectively, and there was no significant difference between them (**Figure 5**).

## Discussion

In the present study, floating leaves were shorter, wider and thicker than submerged leaves in acclimating to the aerial environment. However, the morphology of submerged leaves was longer, narrower and thinner than floating leaves, which increases specific leaf area, enhanced the absorption capacity of DIC in water and decrease water flow resistance in flowing water (Maberly and Gontero, 2018; Li et al., 2019). The anatomical structure results were in line with the previous studies showing that the growth environment affected the morphology and anatomical structure of submerged and floating leaves (Li et al., 2019; Han et al., 2021; Horiguchi et al., 2021; Wang et al., 2022). Besides, the stomata are important for gas exchange and water loss in the atmosphere. The biggest difference between submerged leaves and floating leaves was that floating leaves had stomata on the adaxial leaf surfaces, while submerged leaves had no stomata, indicating that floating leaves have the ability to directly use atmospheric CO_2_ for photosynthesis. The floating leaves can efficiently obtain a stable carbon source through stomata which is one of the development exploitation strategies of aquatic plants to overcome carbon limitation (Maberly and Gontero, 2018; Huang et al., 2020b). This genetic capacity to produce stomata on the upper epidermis of floating leaves is inherited from their terrestrial ancestors and expressed under suitable conditions (Harris et al., 2020) to acclimate to the aerial environment.

Photosynthetic pigments are responsible for light absorption and transformation during photosynthesis (Rosevear et al., 2001). The higher content of Chl-b in submerged leaves is in line with the previous studies showing that the chlorophyll content was higher in submerged leaves than that in aerial leaves in hydrophytes (Johnson et al., 1993; Nekrasova et al., 1998). The ratio of Chl-a/b decreases under light limitation (Dale and Causton, 1992; Ronzhina et al., 2004), which can increase the efficiency of light harvesting at very low irradiation (Rosevear et al., 2001). In the present study, the ratio of Chl-a/b in submerged leaves was lower than that in floating leaves, indicating that submerged leaves better adapted to the underwater shading environment (Baig et al., 2005). Meanwhile, in comparison to the terrestrial plants, the ratio of Chl-a/b was 2.89 in floating leaves that was close to 3 for sunny plants and 2.38 in submerged leaves that was close to 2.3 for shady plants (Lichtenthaler et al., 2007). Both upper and lower epidermal cells containing chloroplasts were presumably beneficial to the submerged leaves absorbing light under a low-irradiance submerged environment. Additionally, transcriptomics studies have found that submerged leaves increase the number of photosynthesis-antenna proteins by increasing the expression of light-harvesting proteins to improve their light-harvesting capacity and better to adapt to the underwater low-light environment (Han et al., 2021). However, in the floating leaves, the absence of chloroplast in the upper epidermal cells could protect against photo-damage under high irradiance. Further, the higher content of anthocyanins also suggested that the floating leaves of *O. ovalifolia* adapted to the strong optical radiation in aerial environments and could quench the free radicals formed by light (Nielsen and Nielsen, 2006). Our present result was in accordance with the results showing that the higher expression of anthocyanins synthetic genes in floating leaves of the heterophyllous plant *Potamogeton wrightii* and which could be responsible for the higher saturated irradiance of floating leaves than that of submerged leaves (Han et al., 2021).

Previous studies reported that ${\text{HCO}}_{3}^{-}$ utilization by aquatic plants provided an additional carbon source for photosynthesis and effectively reduced photorespiration increasing plant primary productivity and growth (Iversen et al., 2019; Li et al., 2021). It was found that ${\text{HCO}}_{3}^{-}$ could be utilized in both floating and submerged leaves of *O. cordata* (Cao et al., 2019; Huang et al., 2020b; Wang et al., 2022), a heteroblastic plant from the same genus. In the present study, the final pH of submerged and floating leaves pH-drift experiment of *O. ovalifolia* was higher than 9.5 indicating that both types of leaves had the ability to use ${\text{HCO}}_{3}^{-}$ (Fernández et al., 2014; Jiang et al., 2021). The value of CT/Alk after the pH-drift experiment could be used to indicate the ${\text{HCO}}_{3}^{-}$ utilization capacity of plants, and the smaller the value, the stronger the utilization capacity (Maberly and Spence, 1983; Madsen and Sand-Jensen, 1991). The lower values of CT/Alk and higher final pH in submerged leaves were similar to previous studies, which suggested that submerged leaves had a stronger ability to use ${\text{HCO}}_{3}^{-}$ than floating leaves. Our present results are in accordance with previous studies on *Potamogeton wrightii* and *O. cordata* (Han et al., 2021; Wang et al., 2022).

In this study, the decrease of the final pH of submerged and floating leaves in AZA treatment indicated that the ${\text{HCO}}_{3}^{-}$ utilization in *O. ovalifolia* depended on the catalytic activity of CAext. This result was consistent with the catalytic utilization of bicarbonate by CAext in submerged plants of the same genus, *O. alismoides* and *O. cordata* (Huang et al., 2020a; Wang et al., 2022). DIDS, an anion exchanger inhibitor, also inhibited the final pH in the pH-drift experiment, indicating that there was a direct absorption of ${\text{HCO}}_{3}^{-}$ by *O. ovalifolia* under our current experimental conditions, which was the same as the result of *O. alismoides* and *O. guanyangensis* (Huang et al., 2020a; Jiang et al., 2021). However, the present result of the DIDS treatment was different from *O. cordata* under DIDS treatment showing that DIDS had no inhibitory effect on the ${\text{HCO}}_{3}^{-}$ use in *O. cordata* (Wang et al., 2022). A previous study reported that submerged plants tended to use ${\text{HCO}}_{3}^{-}$ through the AE pathway only under very low CO_2_ conditions (Axelsson et al., 1995). While the concentration of CO_2_ *in-situ* water was ~55 μM that was much lower than that in *O. cordata* growth conditions (510 μM). In addition, studies on *O. guanyangensis* at different locations in a karst river supplied by underground water found that the plant did not induce AE pathway to use ${\text{HCO}}_{3}^{-}$ at the upstream with a higher concentration of CO_2_, while the AE pathway was induced at the downstream with lower concentrations of CO_2_ (Jiang et al., 2021). Thus, the low concentration of CO_2_ in the growth conditions could be responsible for the direct use of ${\text{HCO}}_{3}^{-}$ *via* AE in the present study.

Besides ${\text{HCO}}_{3}^{-}$ use, C4 and CAM pathways are also important amelioration strategies in submerged plants, which can improve the concentration of CO_2_ at Rubisco active site and adapt to CO_2_ deficiency under water (Madsen and Sand-Jensen, 1991; Klavsen et al., 2011; Maberly and Gontero, 2018; Wang et al., 2022). The slight changes in diel titratable acid of *O. ovalifolia* suggested that both submerged and floating leaves did not perform CAM pathway. It was found that CAM metabolism was induced in *O. alismoides* under high light and low CO_2_ conditions (11 µM), while it then disappeared under high light and high CO_2_ conditions (286 µM) (Shao et al., 2017). Similar results were found in the floating leaves of *O. cordata* research which the CAM appear in low carbon conditions (15 µM) (Huang et al., 2020b). Nevertheless, when the CO_2_ concentration was extremely high in the plants growing environment (510 µM), the CAM was absent in *O. cordata* (Wang et al., 2022). In the present study, the CO_2_ concentration of plant growth water was 55 µM, 3.67-fold higher than that in water at air equilibrium conditions (15 µM), which might be the reason why there was no CAM in *O. ovalifolia*. Meanwhile, floating leaves exposed to air use CO_2_ more easily in the air for photosynthesis and without water deficiency, so the lack of CAM pathway in *O. ovalifolia* was reasonable.

The δ^13^C of atmospheric CO_2_ was usually maintained at about –8‰. When plants used atmospheric CO_2_ as the sole carbon source for photosynthesis, the δ^13^C of C3 plants in the range of –20‰ to –37.5‰ and C4 plants in the range of –10‰ to –17‰ had been reported (Farquhar et al., 1989; von Caemmerer et al., 2014). In our present research, δ^13^C of the floating leaf was –30.07‰, indicating that the floating leaf may perform C3 metabolism and utilize atmospheric CO_2_ as the main carbon source. Different from floating leaves, submerged leaves are completely immersed in water and cannot contact the atmosphere which results in free CO_2_ and ${\text{HCO}}_{3}^{-}$ underwater being their potential carbon sources. In the present study, if free CO_2_ was the only inorganic carbon source, the δ^13^C of C3 and C4 were –37.65‰ and –23.96‰, respectively; if ${\text{HCO}}_{3}^{-}$ was the only inorganic carbon source, the δ^13^C of C3 and C4 were –29.9‰ and –16.11‰, respectively. However, the free CO_2_ concentration in the study water was 55 μM, which was much less than the inorganic carbon of half-saturated photosynthesis (100–200 μM) in submerged plants that force the plant to use heavier ^13^C isotope and affects the isotope fractionation effect (Farquhar et al., 1989), therefore, it cannot be directly concluded that submerged leaves have a C4 pathway through the δ^13^C value. However, based on the enzyme activity analysis, the submerged leaves of *O. ovalifolia* exhibited carboxylase, PEP regeneration enzyme and decarboxylase activities, which are key enzymes in the C4 process. In addition, the PEPC/Rubisco ratio was 2.24 in submerged leaves and 0.988 in floating leaves. The PEPC/Rubisco ratio in typical terrestrial C3 plants and aquatic plants lacking biochemical concentrating mechanisms was generally less than 1 (Zhang et al., 2014). However, previous studies reported that PEPC and PPDK not only contribute to the C4 pathway, but also could participate in other metabolisms in both C4 and C3 plants (Sheen, 1991; Kawamura et al., 1992; Dong et al., 1998; Rao et al., 2008). The different isoforms of PEPC and PPDK genes expressed patterns and posttranslational regulation (Sheen, 1991; Kawamura et al., 1992; Dong et al., 1998; Rao et al., 2008). In the present study, we did not analyze the sequences of PEPC and PPDK or the gene expression of the different isoforms. Thus, the activities of enzymes seemed to imply that submerged leaves might have the ability to perform C4 metabolic pathway, but it needs more evidence to support it in future works. Further, the PEPC/Rubisco ratio, and the δ^13^C isotopic abundance both proved that floating leaves do not have a C4 pathway.

The present study found that *O. ovalifolia* possessed the capacity to use ${\text{HCO}}_{3}^{-}$ and the juvenile leaves of *O. ovalifolia* also could perform the C4 pathway to acclimate to low CO_2_ underwater and at the adult stage, the floating leave of *O. ovalifolia* could directly use CO_2_ from the air, which increases the adaptation range of CO_2_ conditions. It is widely accepted that the ability of plants to access and utilize limiting resources, eventually affecting their competitive advantages, is important for their distribution (James et al., 1999; Cavalli et al., 2012). As for the invasive ability of *O. ovalifolia*, it is still necessary to compare its utilization efficiency of inorganic carbon with that of native species and to understand its reproductive strategy and the mechanism of population formation.

## Conclusions

In the present study, we investigated how an invasive heteroblastic aquatic plant, *O. ovalifolia*, adapts to aquatic and aerial environments. In morphology and anatomical features, submerged leaves are thinner, narrower and longer to increase inorganic carbon absorption in water by increasing specific surface area, while floating leaves directly absorb CO_2_ from the atmosphere through stomata. The difference in chlorophyll and anthocyanin content between submerged and floating leaves is an adaptation to aquatic and aerial environments. There are also differences in CCMs between submerged and floating leaves. Both floating and submerged leaves had the ability to use ${\text{HCO}}_{3}^{-}$ by the external carbonic anhydrase catalysis and the AE pathway. Furthermore, the enzyme results implied that submerged leaves might perform C4 metabolism while floating leaves perform the C3 pathway for photosynthesis. In general, ${\text{HCO}}_{3}^{-}$ utilization, C4 metabolism and CO_2_ absorption are the inorganic carbon utilization strategies for *O. ovalifolia* to cope with the inorganic carbon between aquatic and terrestrial environments, which is conducive to better adaptation to aquatic and terrestrial habitats.

## Data availability statement

The raw data supporting the conclusions of this article will be made available by the authors, without undue reservation.

## Author contributions

HJ and WL designed the experiments. HJ and ZL performed the experiments. ZL analyzed data and wrote the first edition of the manuscript. PL and JZ modified the manuscript. All authors contributed to the article and approved the submitted version.
