# Supplementary material for: Different photosynthetic inorganic carbon utilization strategies in the heteroblastic leaves of an aquatic plant Ottelia ovalifolia
Source: Front Plant Sci. 2023 Mar 24;14:1142848. doi: 10.3389/fpls.2023.1142848 (PMC10081514; doi:10.3389/fpls.2023.1142848)
Supplement: Supplementary file 1 [file DataSheet_1.docx]

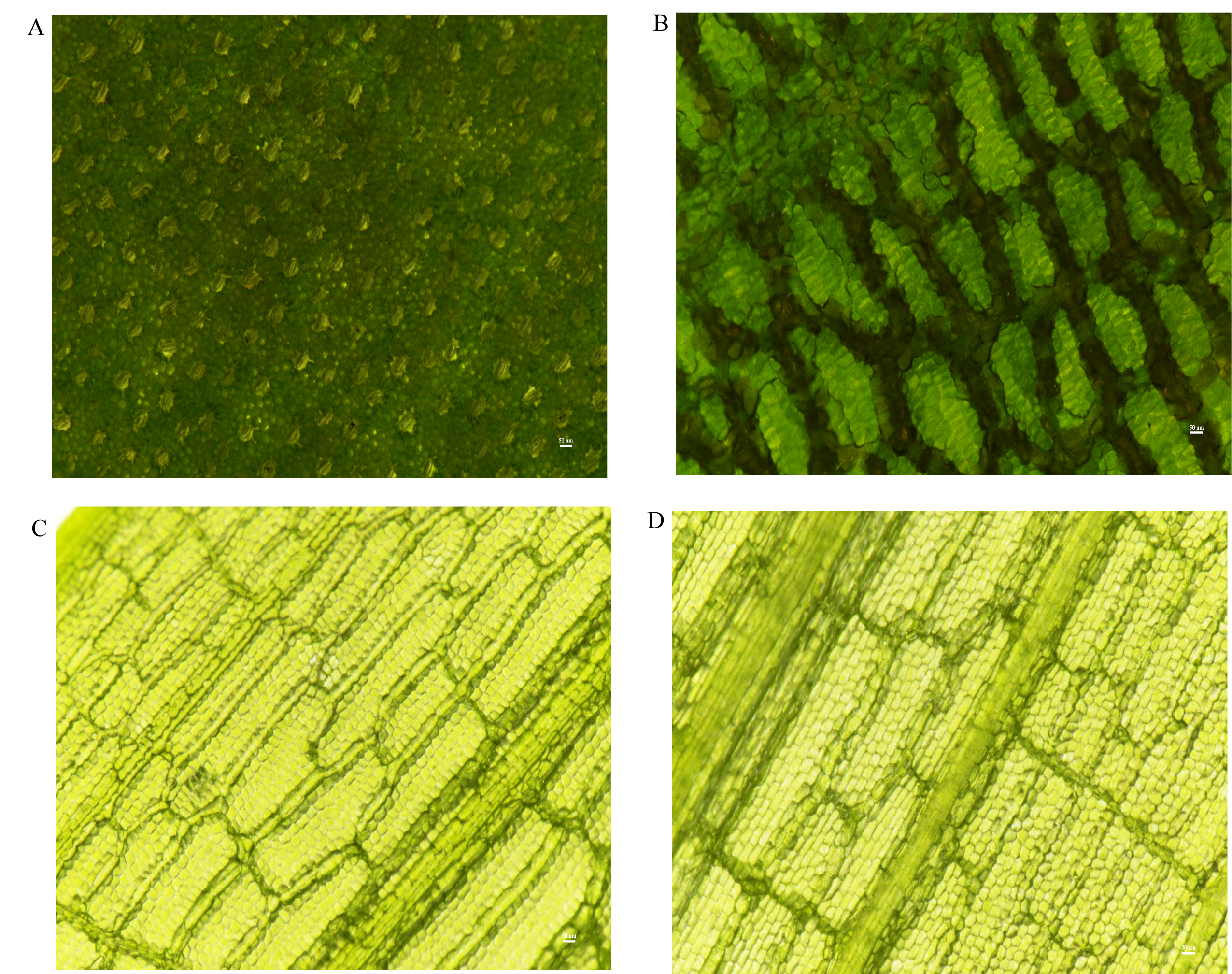


**FIGURE S1|** Distribution of stomata in the blades. (A) Adaxial of floating leaf. (B) Abaxial of floating leaf. (C) Adaxial of submerged leaf. (D) Abaxial of submerged leaf. n=5.
